# Progress Toward Onchocerciasis Elimination in Brazil

**DOI:** 10.4269/ajtmh.23-0749

**Published:** 2024-07-09

**Authors:** João Luiz Pereira de Araujo, Dalila Ríos, Maria Eugenia Grillet, Alda Maria da Cruz, Lindsay Rakers, Frank Richards, Heriberto Francis Schuertz, Sandra Maria Barbosa Durães

**Affiliations:** ^1^ Brazilian Ministry of Health, Secretary of Health and Environment Surveillance, Boa Vista, Brazil;; ^2^ Onchocerciasis Elimination Program for the Americas, Carter Center Guatemala Office, Guatemala City, Guatemala;; ^3^ Laboratorio de Biología de Vectores y Parásitos, Instituto de Zoología y Ecología Tropical, Facultad de Ciencias, Universidad Central de Venezuela, Caracas, Venezuela;; ^4^ Instituto Oswaldo Cruz/FIOCRUZ and Medical School Faculty/Rio de Janeiro State University, Rio de Janeiro, Brazil;; ^5^ The Carter Center, Atlanta, Georgia;; ^6^ Universidade Federal Fluminense, Rio de Janeiro, Brazil

## Abstract

The single onchocerciasis-endemic focus in the remote Amazon rainforest is shared by Brazil and Venezuela and affects primarily the indigenous Yanomami people. Regional elimination of onchocerciasis is challenged by the magnitude and inaccessibility of this area. In Brazil, 272 onchocerciasis-endemic communities are operationally organized through 21 health centers (“polos bases”). Mass drug administration of ivermectin began in 1995, with 36 effective biannual rounds (≥85% coverage of the eligible population) through 2022. The national on chocerciasis program maintains community-level monitoring to prioritize treatment activities and epidemiological surveys. The Onchocerciasis Elimination Program for the Americas and the WHO onchocerciasis elimination guidelines have helped Brazil move toward its goal of stopping ivermectin treatment by 2025 and verifying transmission elimination by 2030. Additional challenges to the Brazilian onchocerciasis program include cross-border movements and insecurity due to illegal mining and inter-community conflicts. The new government in Brazil offers hope given its commitment to the equity of indigenous people and preservation of the Amazon environment.

## INTRODUCTION

Human onchocerciasis is a chronic infection caused by the *Onchocerca volvulus* parasite, which is transmitted through the bites of *Simulium* blackflies that breed in fast-flowing rivers.^1^ This neglected tropical disease is a leading infectious cause of blindness in the world^2^ and is commonly known as river blindness.^2^ In Brazil, onchocerciasis transmission takes place within the indigenous homeland of the Yanomami people, where the Amazonian forest landscape is composed of both low-lying plains and towering mountains in the northernmost states of Roraima and Amazonas.

**Figure 1.**
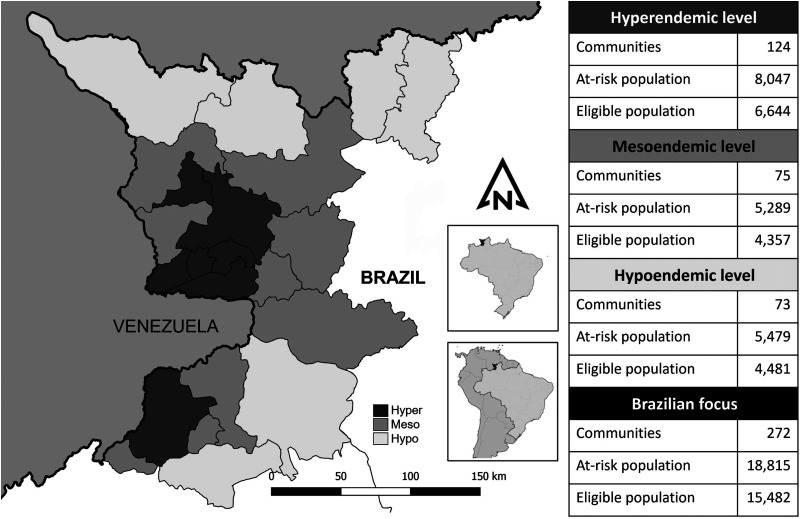
Brazilian onchocerciasis endemic focus demography in 2022 by baseline endemicity. Baseline (pre-treatment) endemicity was classified as follows: hyperendemic (≥60% microfilaridermia prevalence), mesoendemic (20–59%), and hypoendemic (1–19%). Hyper = hyperendemic; Hypo = hypoendemic; Meso = mesoendemic.

The dense forest and rivers with rapids and waterfalls make access to onchocerciasis-endemic Yanomami villages extremely difficult. The highest endemicity occurs in villages located in the Parima Mountains, near the Venezuelan border. In fact, there is a contiguous zone of cross-border disease transmission shared by the Brazilian Focus and Venezuela’s South Focus, and this represents a major challenge for the goal of eliminating onchocerciasis: Transmission must also be interrupted in the neighboring country’s territory to achieve the goal of eliminating the disease in each country. The Brazilian Focus (the country’s only endemic focus) includes 18,815 inhabitants residing in 272 communities organized within a protected national reserve, categorized according to the baseline prevalence of onchocerciasis into hyperendemic (≥60% microfilaridermia prevalence), mesoendemic (20–59%), and hypoendemic (1–19%) areas (Figure 1).^3^

The Yanomami people are the largest indigenous group in the Amazon Region,^4^ preserving their traditional way of life despite substantial outside exposure and pressure.^4^^,^^5^ They are a hunter-gatherer society that is distinct genetically, anthropometrically, and linguistically from neighboring indigenous peoples. They inhabit the Amazon rainforest, covering approximately 74,132 square miles across Brazil and Venezuela, with their territory on the Brazilian side stretching from Roraima’s Branco River basins to the left bank of the Negro River in Amazonas state.^6^^,^^7^ Healthcare for all indigenous populations in the Amazon Region of Brazil is provided by about 280 Ministry of Health staff organized into multidisciplinary health teams that are based in core health centers (“polos bases”) located throughout the region. The teams, which include indigenous members, rotate into and out of the polos bases throughout the year (30 days of service and 15 days off), arriving first by plane or helicopter and from there walking or going by boat to the communities to deliver primary health services.

The elimination strategy of the Onchocerciasis Elimination Program for the Americas (OEPA), which Brazil has adopted, is to eliminate the eye and skin disease produced by *O. volvulus* and interrupt its transmission through effective mass drug administration (MDA) of ivermectin (Mectizan^®^, Merck & Co., Inc., Rahway, NJ) in all endemic areas of the Americas.^8^ Here, ≥85% of the eligible population per treatment round is considered “effective.” Treatment rounds are provided at least twice per year (every 6 months or semiannually). Pregnant women, women in the first week of lactation, persons with chronic debilitating diseases, and young children (<90-cm height, <5 years old, and <15-kg body weight) are not eligible for treatment. Onchocerciasis was formerly prevalent in six countries in the Americas, but with the successful implementation of the OEPA’s strategy, the WHO has verified the elimination of disease in four of them (Colombia, Ecuador, Guatemala, and Mexico), and the only remaining endemic at-risk population resides in the Yanomami Focus Area, composed of the Brazilian Focus and Venezuela’s South Focus.^9^

**Figure 2.**
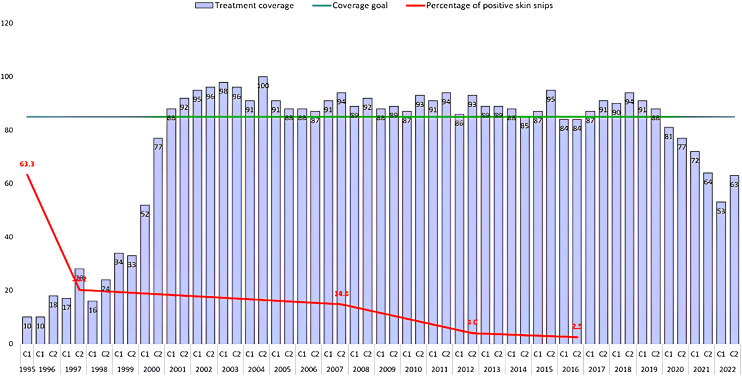
Mass drug administration treatment coverage by year and semiannual cycle (C1, C2) and microfilaridermia prevalence, Brazilian Focus, 1995–2022. Prevalence of microfilaridermia was based on a convenience sample of all age groups. The numbers of persons examined and the number and locations of communities/polo bases sampled are unavailable.

Onchocerciasis treatment in the Brazilian Focus was launched in 1995 with technical support from the OEPA, which has been supporting other program activities in Brazil since 1993.^10^ The treatment rounds have been provided semiannually except from 2011 to 2017, when the Brazilian onchocerciasis program increased to four rounds per year in an effort to accelerate the elimination process. However, the Brazilian onchocerciasis program encountered great logistical difficulties reaching effective coverage in all four rounds, so in 2018 the treatment strategy reverted to semiannual treatment cycles. Thirty-six effective rounds have been recorded in the Brazilian Focus, as shown in Figure 2. Figure 2 also suggests that MDA has had the expected impact of decreasing the prevalence of microfilaridermia among a convenience sample of all age groups from a baseline of 63.3% in 1995 to 2.5% in 2016 (unpublished data, Brazilian Ministry of Health). This decrease reflects the strong microfilaricide effect of ivermectin. From 2019 to 2021, there was a decrease in treatment coverage because of the COVID-19 pandemic’s disruption of programmatic activities,^11^ and in 2022, coverage was low owing to a problem with the importation of ivermectin. As a result, the 85% target has not been met for the past 3 years (Figure 2).

The Brazilian program is actively working to regain effective treatment levels and execute new strategies to enhance program activities in locations where onchocerciasis transmission continues. One approach has been to develop a community-level database. From the beginning, the smallest endemic administrative unit for MDA and epidemiological survey indicators was the polo base, which is the operational unit of primary healthcare in the field. Under the new strategy, MDA and epidemiological indicators have moved from the polo base administrative unit to the community level. Starting in 2013, the Brazilian onchocerciasis program has recorded epidemiological coverage and indicators by community as well as by polo base and overall focus. The Brazilian onchocerciasis program also has historical records by community starting in mid-2010. These data have allowed a calculation of how many communities have achieved a satisfactory cumulative count of effective rounds likely to interrupt transmission, which the OEPA defines as being ≥20. Communities with an accumulated count falling below 10 effective treatment rounds are classified as super-critical, indicating a need for immediate attention and intervention to provide effective rounds in all future MDA cycles. Communities with a count between 11 and 19 cycles are considered critical, requiring close monitoring and support to improve their performance.

The Brazilian onchocerciasis program’s new approach also includes categorizing communities by logistical variables, as shown in Table 1: community population size, means of transportation, distance for health teams, etc. Most communities are small and distant from the health centers and require helicopter access. Local factors (external to the community itself) also affect the performance and achievement of the Brazilian onchocerciasis program’s goals, such as the presence of illegal mining and inter-community conflicts, requiring qualified sociological and anthropological approaches for the region and population’s features. Categorizing communities using variables related to these local factors enables the Brazilian onchocerciasis program to obtain a concise overview, which aids in better directing human and material resources for operational and supervisory purposes. However, the Brazilian onchocerciasis program depends on government policies or inter-institutional assistance to solve problems such as conflicts and illegal mining. The presence of these variables can significantly influence the ability to conduct healthcare interventions in specific communities. In 2022, more than 20,000 illegal gold miners were estimated to be in the Yanomami area; these largely nonindigenous Brazilians can greatly hinder the onchocerciasis program. Miners are usually armed and suspicious of outsiders and hinder health team movements. Illegal mining has severe consequences for the social dynamics of indigenous communities, including alcohol introduction, violent encounters, the introduction of transmissible infectious diseases such as malaria, river contamination with heavy metals, and the destruction of native vegetation. Inter-community conflicts between the Yanomami pose security risks to health teams and may force them from certain areas, thereby disrupting healthcare interventions and reducing treatment coverage. The presence of conflicts also threatens the safety of health professionals, necessitating evacuations until stability has been restored. Identifying affected communities becomes crucial for service planning, presenting challenges in providing essential care.

**Table 1:** Distribution of communities and population by data categories in the Brazilian Focus

Data Category	Communities (*N* = 272)	Population (*N* = 18,815)	Communities (%)	Population (%)
Effective Treatment Rounds*				
>20 rounds	159	11,169	58.5	59.4
11–19	81	5,633	29.8	29.9
0–10	32	2,013	11.8	10.7
Community Population				
1–50	134	3,686	49.3	19.6
51–99	74	5,441	27.2	28.9
100 or More	64	9,688	23.5	51.5
Baseline Endemicity^†^				
Hyperendemic	124	8,047	45.6	42.8
Mesoendemic	75	5,289	27.6	28.1
Hypoendemic	73	5,479	26.8	29.1
Distance for Health Teams				
Far	146	9,821	53.7	52.2
Intermediate	41	2,875	15.1	15.3
Near	85	6,119	31.3	32.5
Means of Transportation				
Walking	96	7,110	35.3	37.8
Boat	60	3,851	22.1	20.5
Aircraft	46	3,318	16.9	17.6
Boat and Walking	21	1,914	7.7	10.2
Aircraft and Walking	49	2,622	18.0	13.9
Cross-Border Migration				
No	199	13,489	73.2	71.7
Yes	73	5,326	26.8	28.3
Illegal Mining Presence				
No	215	15,216	79.0	80.9
Yes	57	3,599	21.0	19.1
Inter-Community Conflicts				
No	156	11,279	57.4	59.9
Yes	116	7,536	42.6	40.1

* Effective rounds mean ≥85% ivermectin coverage of the eligible population.

^†^ Hyperendemic (≥60% microfilaridermia prevalence), mesoendemic (20–59%), and hypoendemic (1–19%).

Because of the longevity and goals of the Brazilian onchocerciasis program and despite the challenges described above, it is very important to launch epidemiological and entomological assessments that follow WHO guidelines for determining when MDA can be safely stopped. These include 1) replacement of microfilaremia monitoring by more sensitive serological testing of children for recent exposure to the parasite by collecting and processing capillary blood-filter paper samples to detect Ov-16 antibodies and 2) vector collections. Some of the preliminary and encouraging serological results from these new evaluations were reported in the Weekly Epidemiological Record in 2023.^12^ Vectors that have been collected are undergoing taxonomic identification to determine the predominant vector species in various subfocus zones, and as recommended by the WHO, molecular processing of blackfly head pools for *O. volvulus* DNA using the O-150 polymerase chain reaction method is ongoing.^9^

With a new government in place that is focusing on respect for indigenous peoples and preservation of the Amazon rainforest, the Brazilian onchocerciasis program is hopeful that in the coming years, there will be new resources, improved security, national and local political commitments, and enhanced program staff morale and determination. The Brazilian onchocerciasis program envisions that it is possible to achieve and sustain high levels of ivermectin MDA treatment coverage to stop MDA throughout the focus in 2025 and achieve verification of elimination by the WHO by 2030, thereby promoting regional equity in public health for the Yanomami.

## References

[1] Rey L, 2013. Bases da Parasitologia Médica (3rd ed.). Ebook. Rio de Janeiro, Brazil: Guanabara Koogan.

[2] Murdoch ME, 2021. Mapping the burden of onchocercal skin disease. Br J Dermatol 184: 199–207. 32302410 10.1111/bjd.19143

[3] Coelho GE Vieira JBF García-Zapata MTA Schuertz JC, 1998. Identifying areas of epidemiological stratification in an onchocerciasis focus in Yanomami territory, Roraima, Brazil [in Portuguese]. Cad Saude Publica 14: 607–611. 9761614 10.1590/s0102-311x1998000300017

[4] Ramos AR, 1984. Ethnic categories of Sanuma thought: Intra and inter-ethnic contrasts [in Portuguese]. Série Antropologia 45: 1–19.

[5] Kopenawa D Albert B, 2015. A Queda do Céu: Palavras de um Xamã Yanomami. São Paulo, Brazil: Companhia das Letras.

[6] Albert B Barbosa RI Ferreira EJG Castellón EG Homem, Ambiente e Ecologia no Estado de Roraima. Manaus, Brazil: INPA.

[7] Albert B Milliken W, 2009. Urihi A – A Terra-Floresta Yanomami. São Paulo, Brazil: ISA.

[8] Blanks J , 1998. The onchocerciasis elimination program for the Americas: A history of partnership. Rev Panam Salud Publica 3: 367–374. 9734217 10.1590/s1020-49891998000600002

[9] World Health Organization , 2020. *Onchocerciasis: Guidelines for Stopping Mass Drug Administration and Verifying Elimination of Human Onchocerciasis: Criteria and Procedures*. Available at: https://apps.who.int/iris/bitstream/handle/10665/204180/9789241510011_eng.pdf?sequence=1. Accessed August 2, 2023. 26913317

[10] Shelley AJ, 2002. Human onchocerciasis in Brazil: An overview. Cad Saude Publica 18: 1167–1177. 12244349 10.1590/s0102-311x2002000500009

[11] World Health Organization , 2021. Progress in eliminating onchocerciasis in the WHO Region of the Americas: Disruption of ivermectin mass drug administration in the Yanomami focus area due to the COVID-19 pandemic. Wkly Epidemiol Rec 96: 477–484.

[12] World Health Organization , 2023. Progress in eliminating onchocerciasis in the WHO Region of the Americas: Advances towards interrupting the transmission of onchocerciasis from the latest preliminary serological assessments conducted in parts of the Yanomami Focus Area, 2018–2022. Wkly Epidemiol Rec 98: 453–457.

